# Supplementary material for: An enduring rapidly moving storm as a guide to Saturn's Equatorial jet's complex structure
Source: Nat Commun. 2016 Nov 8;7:13262. doi: 10.1038/ncomms13262 (PMC5105178; doi:10.1038/ncomms13262)
Supplement: Supplementary Information — Supplementary Figures 1-3 and Supplementary Tables 1-2 [file ncomms13262-s1.pdf]

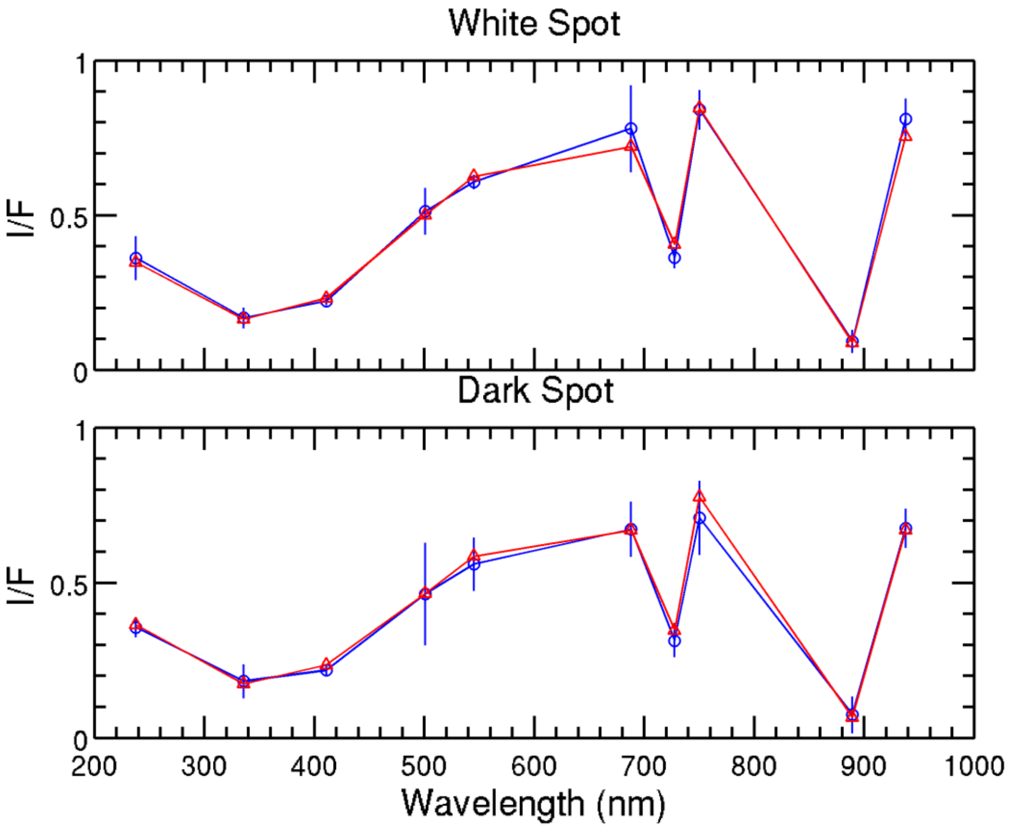

Supplementary Figure 1. Best fitting models (red triangles) for the White and Dark Spots compared with actual observations (blue circles). Error bars result from one standard deviation of the data collected at each filter images.

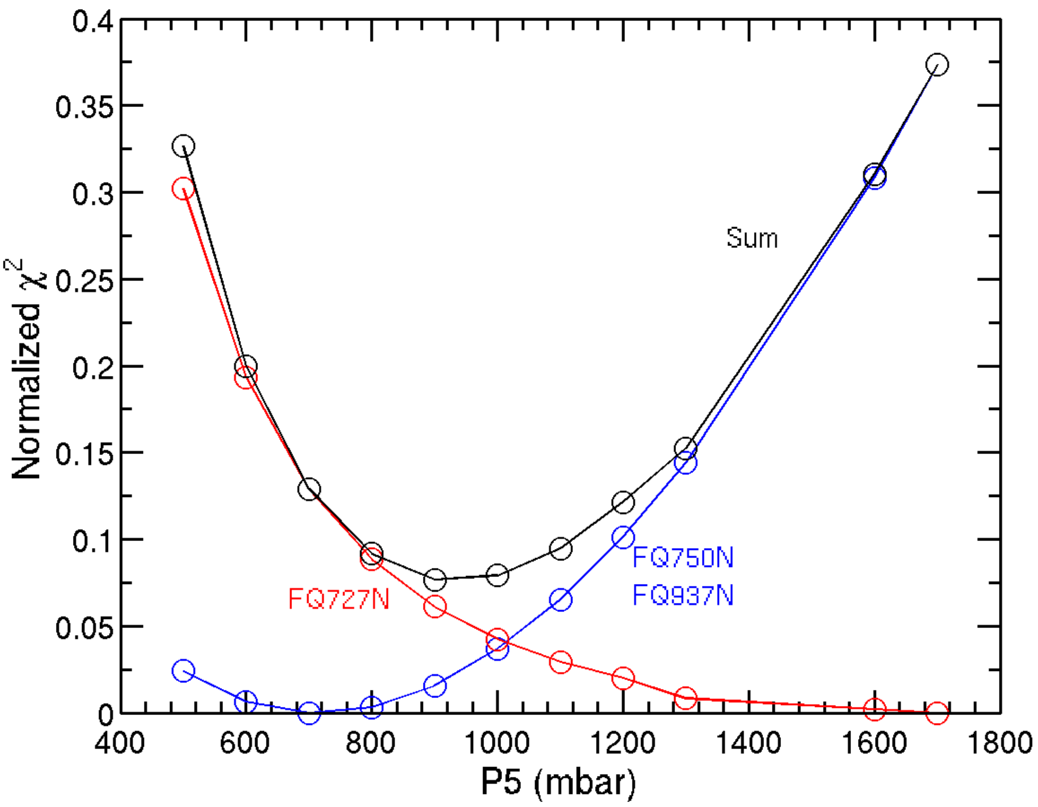

Supplementary Figure 2. Sensitivity of the White Spot model to the pressure level of the bottom cloud. The vertical represents the mean square deviation values normalized to the minimum case for the penetrating filters FQ750N and FQ937N (blue circles), the intermediate FQ727N (red circles) and the sum of all of them. While penetrating filters support a cloud located higher in the troposphere, it would become too bright in FQ727N. A compromise solution is found around 1 bar.

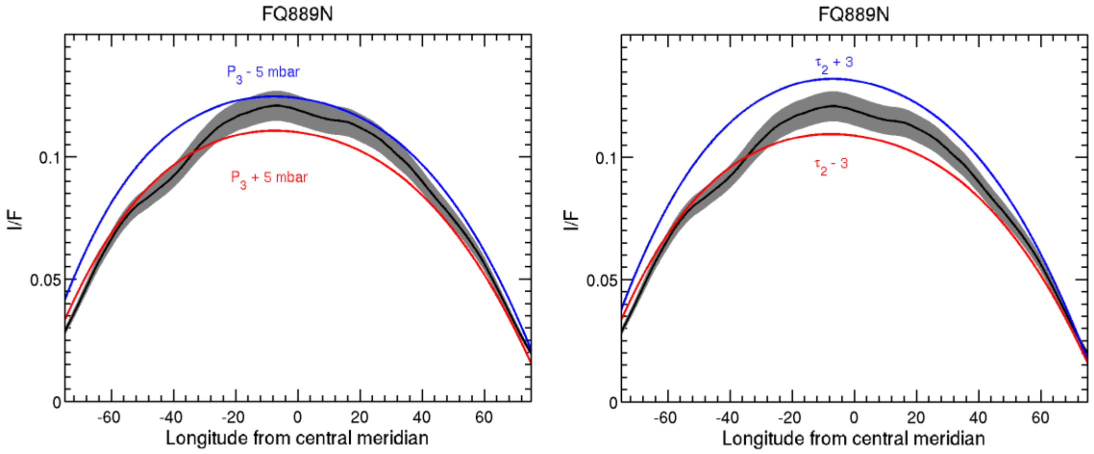

Supplementary Figure 3. Sensitivity of the FQ889N observations (solid line) to the pressure top and optical thickness of the tropospheric haze around the optimum values. Gray area define the calibration uncertainty as described in the text. The fluctuations of the observations around the mean values roughly correspond to changes in the atmospheric parameters shown in this figure.

Supplementary Table 1: Ground-based images, contributors and telescopes

Observer	Location	Telescope diameter	Number of Images
T. Barry*	Australia	40.6 cm	17
T. Olivetti*	Thailand	41 cm	9
M. Kardasis*	Greece	35.6 cm	5
A. Germano*	Brazil	28 cm	4
A. Wesley*	Australia	40.6 cm	4
P. Miles†	Australia	35.6 cm	3
M. Delcroix*	France	32 cm	1
C. Go*	Phillipines	35.6 cm	1
T. Horiuchi†	Japan	30 cm	1
P. Maxson†	USA	31.5 cm	1
**UPV/EHU team***			
A. Sánchez-Lavega	Spain	28 cm (1)	5
Planetcam	Spain	2.2 m (2)	5

***IOPW/PVOL database:**
http://www.pvol.ehu.es/pvol/

†ALPO Japan:
http://alpo-j.asahikawa-med.ac.jp/Latest/Saturn.htm

(1) Aula EspaZio Gela Observatory [29]
(2) Calar Alto Observatory (Centro Astronómico Hispano Alemán), [30]

Supplementary Table 2: HST list of images

Image	Time (UT)	Filter	Exposure (s)
**Date: 29 June 2015**			
ict901kiq_drz.fits	22:56:01	F689M	1.70
ict901kjq_drz.fits	22:57:32	F547M	1.90
ict901kkq_drz.fits	22:58:55	F410M	20.0
ict901klq_drz.fits	23:01:32	FQ727N	40.0
ict901kmq_drz.fits	23:06:21	FQ750N	20.0
ict901knq_drz.fits	23:10:58	FQ889N	55.0
ict901kpq_drz.fits	23:19:56	FQ889N	55.0
ict901kqq_drz.fits	23:25:00	FQ937N	45.0
ict901krq_drz.fits	23:30:18	F502N	23.0
ict901ksq_drz.fits	23:32:35	F225W	55.0
ict901kuq_drz.fits	23:37:27	F225W	55.0
ict901kvq_drz.fits	23:40:19	F336W	30.0
ict901kwq_drz.fits	23:42:03	F689M	1.70
**Date: 30 June 2015**			
ict902u8q_drz.fits	19:36:41	F410M	20.0
ict902u9q_drz.fits	19:39:18	FQ727N	40.0
ict902uaq_drz.fits	19:44:07	FQ750N	20.0
ict902ubq_drz.fits	19:48:44	FQ889N	55.0
ict902ucq_drz.fits	19:53:13	FQ889N	55.0
ict902udq_drz.fits	19:57:42	FQ889N	55.0
ict902ueq_drz.fits	20:02:46	FQ937N	45.0
ict902ufq_drz.fits	20:08:00	F689M	1.70
ict902ugq_drz.fits	20:09:29	F502N	23.0

ict902uhq_drz.fits	20:11:46	F225W	55.0
ict902uiq_drz.fits	20:14:12	F225W	55.0
ict902ukq_drz.fits	20:19:30	F336W	30.0
ict902ulq_drz.fits	20:21:20	F547M	1.90
ict902umq_drz.fits	20:22:39	F689M	1.70
**Date: 1 July 2015**			
ict903auq_drz.fits	17:52:49	F689M	1.70
ict903avq_drz.fits	17:54:18	F502N	23.0
ict903awq_drz.fits	17:56:35	F225W	55.0
ict903axq_drz.fits	17:59:01	F225W	55.0
ict903ayq_drz.fits	18:01:27	F225W	55.0
ict903azq_drz.fits	18:04:19	F336W	30.0
ict903b0q_drz.fits	18:06:03	F689M	1.70
ict903b1q_drz.fits	18:08:35	FQ937N	45.0
ict903b2q_drz.fits	18:13:29	FQ889N	55.0
ict903b3q_drz.fits	18:17:58	FQ889N	55.0
ict903b4q_drz.fits	18:22:27	FQ889N	55.0
ict903b5q_drz.fits	18:27:48	FQ750N	20.0
ict903b6q_drz.fits	18:32:17	FQ727N	40.0
ict903b7q_drz.fits	18:37:31	F547M	1.90
